# Loneliness, rumination, and adolescent psychological crisis in China: a pilot moderated mediation study

**DOI:** 10.3389/fpsyt.2026.1836083

**Published:** 2026-07-09

**Authors:** Huifang Cheng

**Affiliations:** Department of General Education Research, Zhengzhou Police University, Zhengzhou, China

**Keywords:** adolescent mental health, China, loneliness, moderated mediation, pilot study, psychological crisis, resilience, rumination

## Abstract

**Background:**

Adolescent psychological crisis—encompassing depressive disorder, anxiety disorder, and suicidal ideation—is a major public health challenge. One in five adolescents experiences a clinically significant mental health condition globally. Loneliness is a well-documented, modifiable risk factor for these outcomes. However, the exact cognitive-emotional pathways linking loneliness to acute psychological crisis remain unclear. This is particularly true in China, where rapid social change and intense academic pressure increasingly strain adolescent mental health. We do not yet fully understand how protective resources might buffer these specific pathways.

**Objectives and methods:**

This pilot study tests a moderated mediation model based on the Evolutionary Theory of Loneliness and the stress-vulnerability framework. We investigate ruminative thinking as a cognitive-emotional mediator between loneliness and psychological crisis. We also test whether perceived social support and psychological resilience moderate distinct stages of this pathway. Based on a pilot sample of *N* = 312 Chinese adolescents (ages 12–18), we use structural equation modelling (SEM), confirmatory factor analysis (CFA), and Hayes’ PROCESS Macro (Model 14) with 5, 000 bootstrap replications. Two-wave longitudinal data (*n* = 187, 8-week interval) were available for the depression outcome only; the anxiety and suicidal-ideation pathways were examined cross-sectionally.

**Results:**

Loneliness is significantly associated with depressive symptoms (β = 0.43, *p* < 0.001), anxiety (β = 0.38, *p* < 0.001), and suicidal ideation (β = 0.27, *p* < 0.001). Ruminative thinking accounts for 40–46% of the total indirect association across all three outcomes. Social support buffers the transmission from loneliness to rumination (*b* = −0.16, *p* = 0.002, Δ*R*^2^ = 0.023), while resilience buffers the transmission from rumination to crisis (*b* = −0.19, *p* < 0.001, Δ*R*^2^ = 0.031).

**Conclusions:**

These preliminary, exploratory findings are consistent with the possibility that ruminative thinking partially accounts for the cross-sectional association between loneliness and adolescent psychological crisis in China, and with social support and resilience attenuating this association at potentially distinct stages. Because the pilot is underpowered for the moderated mediation model and the available longitudinal evidence covers depression only, the moderation and stage-specificity results should be read as hypothesis-generating rather than confirmatory. A planned full-scale study (*N* ≥ 1, 200) will test these mechanisms further. We also outline initial practical implications for school-based interventions.

## 1 Introduction

The mental health of adolescents has deteriorated substantially across the industrialised and rapidly developing world over the past two decades. The Global Burden of Disease study estimates that mental and substance-use disorders account for nearly 45% of disability-adjusted life-years among individuals aged 10–24. Depression and anxiety disorders alone constitute the leading causes of years lived with disability in this age group (1). In China, national surveillance data indicate that the 12-month prevalence of depressive symptoms among secondary-school students has risen to approximately 24.6%, with suicidal ideation affecting an estimated 14–17% of the adolescent population (2). These figures represent an extraordinary developmental burden, as mental health problems emerging during adolescence are closely tied to long-term adaptation and adult functioning (3).

Against this backdrop, loneliness—the subjective experience of a discrepancy between desired and achieved social connectedness (4)—is a well-established and modifiable risk factor for psychological distress. A meta-analysis of 91 studies by Banerjee and Rai (5) found a robust association between loneliness and depressive symptoms (*r* = 0.47) that persisted even after accounting for objective social isolation, neuroticism, and prior depression. The COVID-19 pandemic further highlighted the health consequences of enforced social disconnection. Longitudinal evidence shows that loneliness onset during pandemic restrictions predicted clinically significant increases in depression and anxiety up to 18 months later (6). Consequently, public health models increasingly emphasise preventive and interventionoriented responses across schools, services, and communities (7, 8).

Specific structural conditions in China amplify the risk that loneliness poses to adolescents. Intense academic pressure and the uneven implementation of school-based mental-health initiatives frequently compound social stress and limit opportunities for timely psychological support (1, 9). Meanwhile, family relationships remain the core of the adolescent social ecology; lower family cohesion is consistently linked to greater distress (10). Finally, while digital socialisation expands opportunities for contact, it does not reliably protect against loneliness and often coexists with problematic smartphone use (11). Despite these clear structural risks, we still know relatively little about the specific pathways linking loneliness to psychological crisis in Chinese adolescents compared to Western samples (9).

This pilot investigation addresses these gaps by evaluating a unified statistical model of loneliness and psychological crisis. Although researchers know loneliness correlates with depression, anxiety, and suicidal ideation, studies rarely examine the cognitive-emotional pathways that account for these associations simultaneously. Ruminative thinking—the tendency to repetitively focus on distressing feelings and their causes (12)—is a theoretically plausible mediating variable. While initial evidence supports a close coupling between loneliness and rumination (13, 14), the complete pathway has not been tested against all three crisis outcomes within a single framework. We also lack a clear understanding of when and how protective resources moderate this risk in Chinese samples. Social support and psychological resilience are both associated with reduced psychopathology, but whether they buffer the risk at distinct points in the pathway remains unclear.

To bridge this specific empirical gap, we present a moderated mediation analysis examining the direct associations of loneliness with depression, anxiety, and suicidal ideation in a Chinese adolescent pilot sample. We test ruminative thinking as a mediating mechanism, and explore perceived social support and psychological resilience as moderators at distinct stages of the distress cascade. Because this is a pilot study, all findings are exploratory; they serve to calibrate a planned, fully-powered confirmatory analysis (*N* ≥ 1, 200).

## 2 Theoretical framework and hypotheses

### 2.1 The evolutionary theory of loneliness and psychological crisis

The Evolutionary Theory of Loneliness (ETL; Cacioppo and Cacioppo 3) provides the primary theoretical architecture for the present study. The ETL conceptualises loneliness not as a peripheral emotional state but as a biological signal, analogous to hunger or pain, that evolved to motivate social reconnection by activating heightened vigilance to social threat. When this vigilance becomes chronic and is not resolved by successful reconnection, it initiates a cascade of neurobiological, cognitive, and behavioural changes that collectively increase vulnerability to psychiatric disorder. The ETL hypothesises that these changes are mediated by cognitive processes. Loneliness shifts attentional resources toward threat-relevant social information (15), increases negative social evaluation expectations (16), and sustains ruminative processing of interpersonal rejection episodes (12).

The stress-vulnerability model (17) complements the ETL by specifying the conditions under which lonelinessrelated stress is associated with clinical disorder. The model distinguishes between stress appraisal (the initial perception of a stressor as threatening) and coping resources (personal and social assets that regulate the stress response). Within this framework, loneliness amplifies stress appraisal through the hypervigilance mechanism described by the ETL, while social support and resilience serve as coping resources that interrupt the stress-to-disorder pathway.

Three theoretical considerations specify how we link these frameworks. The first concerns the process through which loneliness is expected to activate rumination. Under the ETL, the perceived discrepancy between desired and achieved connection places the individual in a state of implicit hypervigilance for social threat: attention is preferentially allocated to rejection-relevant cues (15), ambiguous social information is interpreted negatively, and memories of social failure are encoded and retrieved more readily (16). Because the underlying social deficit is rarely resolved by these cognitive operations, the discrepancy persists as a salient, unattained, self-relevant goal. Theories of perseverative cognition hold that precisely such unresolved self-relevant discrepancies sustain recurrent, intrusive processing of their causes and implications (18): the lonely adolescent repeatedly revisits why overtures failed, what the isolation says about the self, and what its consequences will be. Loneliness thus supplies both the activating condition (an unresolved threat to a fundamental social goal) and the thematic content (perceived social inadequacy) of ruminative episodes, a coupling reinforced by the self-perpetuating regulatory loop in which anticipated rejection suppresses the very reconnection behaviours that would terminate the discrepancy (19).

The second consideration concerns why rumination, rather than another cognitive process, is positioned as the primary candidate mechanism. We do not claim that rumination is the only cognitive route from loneliness to disorder; attentional bias, negative interpretation, and hopelessness are plausible companion processes (16). Rumination is nevertheless the strongest single candidate for three reasons. First, it is transdiagnostic: prospective evidence links ruminative response styles not only to depression but also to anxiety and to self-harm-related outcomes (18, 20), matching the three-outcome crisis construct examined here, whereas worry is predominantly anxiety-specific and future-oriented and hopelessness is most closely tied to depression and suicidality. Second, its content matches the stressor: rumination is past- and present-focused processing of the causes and meanings of distress, which corresponds directly to the unresolved social deficit that defines loneliness, and network analyses accordingly identify loneliness and rumination as adjacent, mutually reinforcing nodes in adolescent symptom networks (13). Third, direct mediation evidence already exists for the depression pathway (12), providing an empirical anchor that the present study extends to anxiety and suicidal ideation.

The third consideration concerns the rationale for dividing the pathway into two stages. The transactional model of stress (21) separates the appraisal of a stressor from the coping processes mobilised by that appraisal, and the stressbuffering hypothesis (22) explicitly identifies two distinct points at which protective resources can intervene: between the stressor and the appraisal-driven stress response, and between that response and its pathological outcome. The present two-stage structure maps the loneliness–rumination–crisis cascade onto these two intervention points. Stage 1 is the transition from stressor to perseverative appraisal (loneliness → rumination); Stage 2 is the transition from perseverative appraisal to clinical outcome (rumination → crisis). The assignment of resources to stages follows their functional character: perceived social support is an external, appraisal-relevant resource that signals the actual availability of connection and should therefore undercut the deficit appraisal before it becomes perseverative (17, 22), whereas resilience is an internal regulatory capacity that is deployed after negative cognition has arisen, interrupting its escalation into disorder (3). We emphasise that the two-stage division is an analytic simplification rather than a claim of exclusivity; each resource may also operate, more weakly, at the other stage, and this possibility is examined empirically in the sensitivity analyses (Section 4).

Goossens (23) extended the ETL specifically to adolescent populations, arguing that the developmental tasks of adolescence (identity formation, peer-group belonging, and the renegotiation of family relationships) make adolescents particularly susceptible to the threat-hypervigilance processes described by the theory. Adolescents who experience chronic loneliness may enter a regulatory loop (19) in which anticipated social rejection suppresses prosocial behaviour, further reducing social connection and deepening loneliness in a self-perpetuating cycle. This loop is theoretically amplified during the heightened social sensitivity of early-to-middle adolescence, consistent with developmental data showing that loneliness peaks around ages 13–14 and again in late adolescence (8).

### 2.2 Ruminative thinking as a mediating mechanism

Ruminative thinking refers to a perseverative, self-focused cognitive style in which individuals repeatedly return to the causes, meanings, and consequences of their negative emotional experiences without moving toward problem resolution (12). Within the Response Styles Theory (12), rumination is conceptualised as a maladaptive emotion regulation strategy that prolongs and intensifies negative affect, thereby increasing vulnerability to clinical depression. A response-styles perspective on loneliness suggests that the perceived social deficit activated by loneliness provides the focal content for ruminative episodes: lonely individuals ruminate over rejected overtures, social inadequacy, and the painful contrast between current isolation and desired connection.

Recent network analysis identifies loneliness and rumination as adjacent, mutually reinforcing nodes in adolescent symptom networks (13), while Mathers et al. (12) showed that uncontrollable ruminative thoughts mediate the association between loneliness and depressive symptoms. However, no study has tested rumination as a mediator of the full loneliness-to-psychological-crisis association spanning depressive symptoms, anxiety, and suicidal ideation simultaneously.

We therefore test the following baseline and mediation hypotheses:

H1: Loneliness is positively associated with depressive symptoms (H1a), anxiety (H1b), and suicidal ideation (H1c) among Chinese adolescents.
H2: Loneliness is positively associated with ruminative thinking.
H3: Ruminative thinking positively mediates the associations between loneliness and depressive symptoms (H3a), anxiety (H3b), and suicidal ideation (H3c).

### 2.3 Perceived social support as a stage-1 moderator

The Social Support Buffering Hypothesis (17) predicts that social support mitigates the deleterious effects of stressors by providing emotional, informational, and tangible resources that reduce the perceived threat of those stressors. We expect social support to buffer this process at Stage 1. By signalling actual social availability, high social support should suppress the activation of rumination. This prediction is consistent with evidence that perceived social integration attenuates hypervigilance responses in socially isolated individuals (24) and that interpersonal support reduces trait rumination in longitudinal studies (25).

In Chinese adolescents, social support is particularly relevant given the cultural emphasis on collectivist relational obligations and the psychological salience of family and peer belonging. Tang et al. (26) found that perceived social support from family, peers, and teachers each independently predicted reduced depressive symptoms among Chinese secondary-school students. Family cohesion has also been conceptualised as a protective factor against adolescent loneliness and psychological distress, suggesting that supportive family environments weaken the emotional consequences of social disconnection (10). We therefore hypothesise:

H4: Perceived social support moderates the association between loneliness and rumination, such that the positive loneliness-to-rumination association is weaker for adolescents with higher social support.

### 2.4 Psychological resilience as a stage-2 moderator

Psychological resilience is broadly defined as the capacity for positive adaptation in the context of significant adversity or risk (3). We expect psychological resilience to moderate the pathway at Stage 2, between ruminative thinking and psychological crisis outcomes. High-resilience adolescents possess more flexible cognitive emotion regulation strategies that can interrupt ruminative chains before they reach a clinical threshold (3). Longitudinal evidence supports this prediction: Zhang et al. (27) found that resilience moderated the prospective effect of negative life events on depression over a 12-month period, an effect that was fully mediated by reduced ruminative response style.

Leve et al. (28) proposed a three-factor model of adolescent resilience (positive emotions, problem-focused coping, and meaning- making) that is consistent with the present Stage-2 moderation hypothesis, because all three components are plausibly activated by high-resilience adolescents when confronted with ruminative cognition. In China, resilience has been identified as a key target in national school mental health curricula (29), and the Connor–Davidson Resilience Scale has demonstrated robust psychometric properties in Chinese adolescent samples (3). We therefore hypothesise:

H5: Psychological resilience moderates the association between rumination and depressive symptoms, such that the positive rumination-to- depression association is weaker for adolescents with higher resilience.
H6: Psychological resilience moderates the association between rumination and anxiety, such that the positive rumination-to-anxiety association is weaker for adolescents with higher resilience.
H7: Psychological resilience moderates the association between rumination and suicidal ideation, such that the positive rumination-to- suicidal-ideation association is weaker for adolescents with higher resilience.

**Figure 1:**
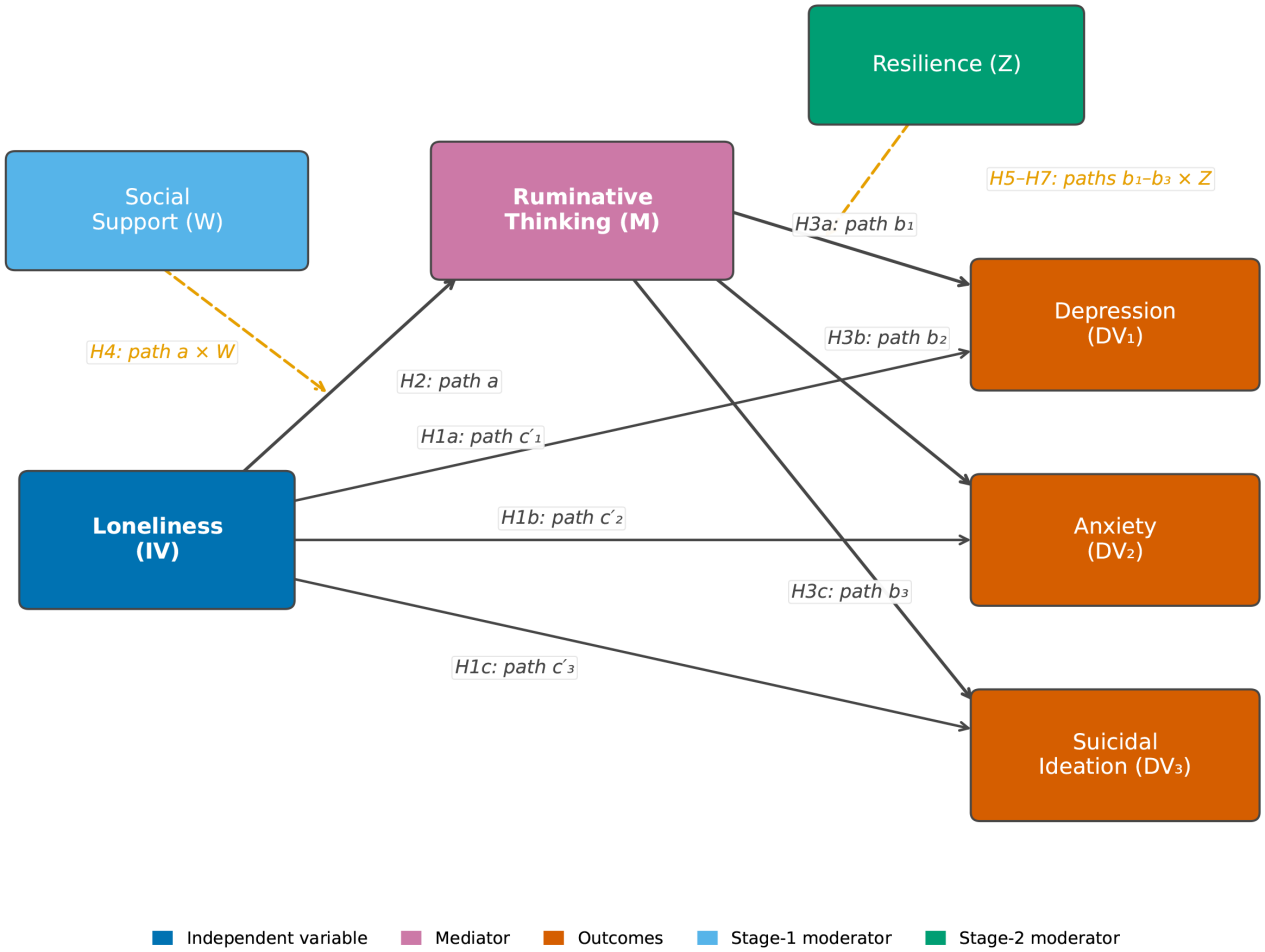
Proposed moderated mediation framework. Loneliness (IV) predicts the three psychological crisis outcomes both directly (H1a–H1c: paths $c_{1}^{'}–c_{3}^{'}$, to depression, anxiety, and suicidal ideation, respectively) and indirectly via ruminative thinking (M), through the combination of H2 (path *a*) and H3a–H3c (paths *b*_1_–*b*_3_). Perceived social support (W) moderates the loneliness-to-rumination path (Stage 1; H4: path *a* × *W*). Psychological resilience (Z) moderates the three rumination-to-outcome paths (Stage 2; H5–H7: paths *b*_1_×*Z*, *b*_2_×*Z*, and *b*_3_×*Z*). Every structural path in the diagram carries its hypothesis number and path label.

Together, Hypotheses H2 through H7 specify a moderated mediation model in which social support and resilience jointly attenuate the indirect effect of loneliness on psychological crisis via rumination. The complete theoretical framework is depicted in **Figure 1**.

## 3 Methods

### 3.1 Participants and sampling design

This paper reports findings from a pilot study (*N* = 312) conducted to evaluate the feasibility of the measurement instruments, assess model identification, and obtain preliminary effect size estimates. A larger confirmatory study is planned; the present manuscript reports only the pilot findings. No data from the planned full-scale study are available at the time of submission.

The pilot subset was drawn from de-identified records archived in the Chinese National Survey Data Archive (CNSDA). In the source survey, respondents had been sampled from four middle schools and two senior high schools selected via stratified cluster sampling, stratified by school type (public vs. private), urban–rural location, and regional GDP quintile. The records retained for the present analysis were those of respondents who were (a) enrolled in Grades 7–12, (b) aged 12–18 years, (c) able to read Mandarin Chinese, and (d) not recorded as undergoing inpatient psychiatric treatment; records flagged in the archive for severe cognitive impairment or active psychotic symptoms were excluded. No direct human-subject research was conducted in the present study: the analysis exclusively used de-identified, publicly available secondary data, and the source survey investigators were responsible for participant recruitment, consent, and screening. On this basis the present secondary analysis was exempt from institutional ethics committee review under standard guidelines for research with de-identified public data.

**Figure 2:**
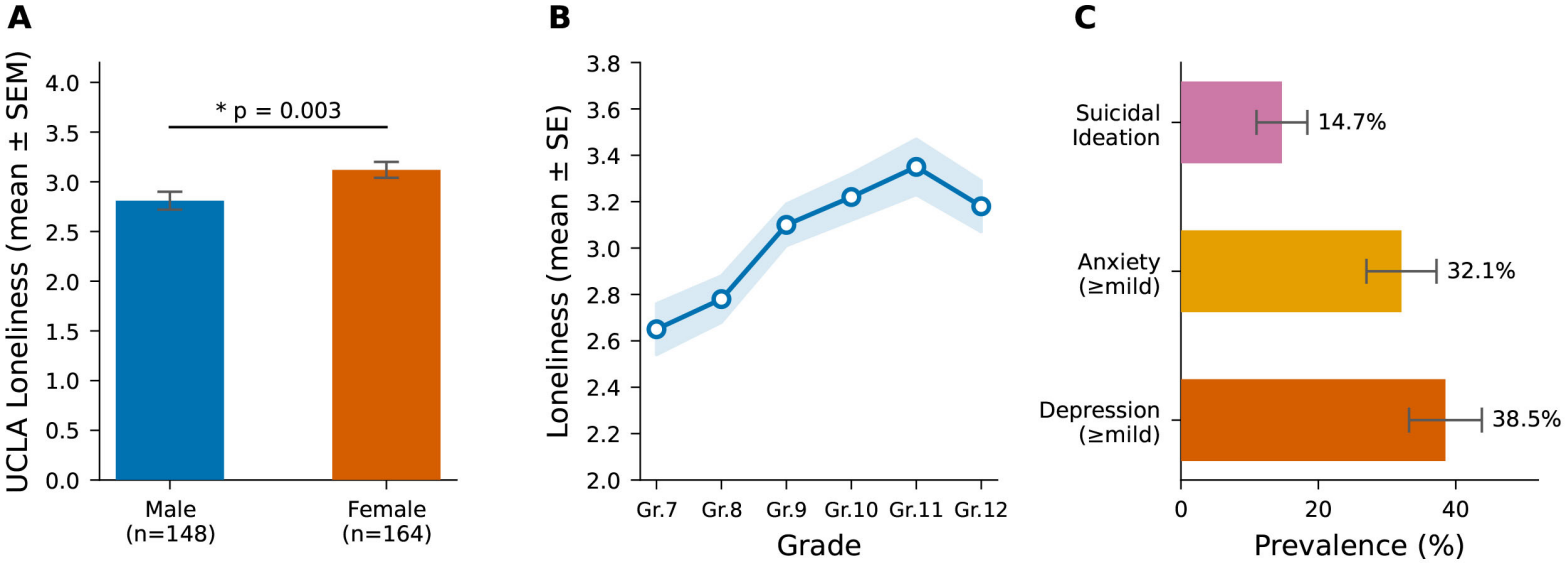
Pilot sample descriptive statistics. **(A)** Mean UCLA Loneliness scores by gender (error bars = ± 1 SEM; asterisk indicates *p* = 0.003). **(B)** Loneliness scores across grade levels with 95% confidence band. **(C)** Prevalence of clinical cut-off crisis outcomes (error bars = 95% CI). Dep, depression (PHQ-9 ≥ 5); Anx, anxiety (GAD-7 ≥ 5); Sui, suicidal ideation (C-SSRS-SR active ideation at least weekly).

The pilot sample (*N* = 312; 52.6% female; *M*_age_ = 14.8, *S D* = 1.7; range 12–18) was recruited during the 2023–2024 academic year. **Table 1** provides full demographic characteristics. Pilot descriptive statistics and prevalence estimates are depicted in **Figure 2**.

**Table 1:** Pilot sample demographic characteristics (*N* = 312).

Variable	Category	*n*	%
Gender	Male	148	47.4
	Female	164	52.6
Grade Level	Grade 7	48	15.4
	Grade 8	52	16.7
	Grade 9	56	17.9
	Grade 10	58	18.6
	Grade 11	55	17.6
	Grade 12	43	13.8
Hukou Type	Urban	189	60.6
	Rural	123	39.4
Family Structure	Two-parent	251	80.4
	Single-parent	38	12.2
	Other	23	7.4

### 3.2 Measures

All measures were administered in Mandarin Chinese via paper-and-pencil questionnaire. Where available, validated Chinese translations were used; where only the original English scale was available, standard back-translation procedures (translation by two bilingual psychologists, independent back-translation, reconciliation panel) were applied.

Loneliness. Loneliness was assessed using the 20-item UCLA Loneliness Scale (Version 3; Cacioppo and Hawkley 5). Items are rated on a 4-point scale from *never* (1) to *always* (4). The scale has demonstrated excellent reliability and validity in Chinese adolescent samples, with Cronbach’s α typically exceeding 0.88 (30). In the pilot sample, α = 0.91, mean inter-item *r* = 0.34.

Depression. Depressive symptoms were measured with the 9-item Patient Health Questionnaire [PHQ-9; 35]. Each item is rated on a 4-point Likert scale (0 = *not at all* to 3 = *nearly every day*); a total score ≥ 5 indicates mild-to-severe depressive symptoms. The Chinese PHQ-9 has been validated in adolescent samples and shows strong sensitivity and specificity against structured diagnostic interviews (2). Pilot α = 0.87.

Anxiety. Anxiety was assessed with the 7-item Generalised Anxiety Disorder Scale [GAD-7; 8], rated on the same 4-point Likert format (≥ 5 = mild anxiety or above). The Chinese GAD-7 has demonstrated strong convergent validity with the State-Trait Anxiety Inventory in secondary-school samples (17). Pilot α = 0.85.

Suicidal Ideation. Suicidal ideation was measured with the 19-item Columbia Suicide Severity Rating Scale SelfReport version (C-SSRS-SR; Posner et al., 2011), which provides a dimensional score and categorical classification of ideation intensity. The Chinese C-SSRS-SR has been validated in adolescent inpatient and community samples (31). We report both the continuous ideation intensity score and the proportion endorsing at least weekly active ideation.

Ruminative Thinking. Rumination was assessed with the 10-item Ruminative Response Scale (RRS-10; Mathers et al., 29), comprising two subscales: brooding (passive comparison of current state with better states) and reflective pondering (purposeful self-reflective attention). Brooding shows consistently stronger associations with depression and is reported separately in supplementary analyses. Pilot α = 0.83.

Perceived Social Support. Social support was assessed with the 12-item Multidimensional Scale of Perceived Social Support [MSPSS; 37], which yields subscale scores for family, friend, and significant-other support. The Chinese MSPSS has demonstrated strong three-factor structure and criterion validity in adolescent samples (26). Pilot α = 0.90.

Psychological Resilience. Resilience was assessed with the 10-item Connor–Davidson Resilience Scale [CDRISC-10; 6]. The broader Connor–Davidson Resilience Scale has shown acceptable psychometric properties in Chinese samples (32). Pilot α = 0.88.

Covariates. Age, gender, grade level, hukou type (urban/rural), family structure (two-parent vs. single-parent), and family socioeconomic status (SES; assessed via parental education and household income bracket) were included as covariates in all models.

### 3.3 Confirmatory factor analysis

Prior to testing the structural model, we conducted a measurement model (CFA) to evaluate the factorial validity of the six primary constructs simultaneously. A six-factor model was specified with parcels for loneliness and rumination and individual items for depression, anxiety, suicidal ideation, social support, and resilience. Parceling was applied to loneliness (UCLA-20, 20 items) and rumination (RRS-10, 10 items) using the item-to-construct balance method (33), which groups items into composite indicators to reduce model complexity and improve indicator reliability for longer scales while preserving latent-variable estimation of measurement error. Three parcels were created for each of these two constructs. Items from shorter scales (PHQ-9, GAD-7, MSPSS, CD-RISC-10) were retained at the item level given their more manageable length. As a sensitivity check, the structural model was re-estimated with item-level indicators for loneliness and rumination; key path coefficients changed by less than 0.03, supporting the robustness of the parceling approach. We evaluated model fit using the comparative fit index (CFI), Tucker- Lewis index (TLI), root mean square error of approximation (RMSEA), and standardised root mean square residual (SRMR), applying the criteria recommended fit criteria: CFI/TLI > 0.95, RMSEA < 0.06, and SRMR < 0.08 (3). All structural models were estimated in R (Version 4.3) using the lavaan package (Version 0.6) with maximum likelihood estimation and robust standard errors (MLR) to account for non-normality.

### 3.4 Structural equation modelling

The structural model was estimated in two stages. In Stage A, the direct and indirect pathways (H1–H3) were estimated simultaneously within the lavaan SEM framework. Indirect effects (loneliness → rumination → outcome) were evaluated using 5, 000 bootstrap replications to generate 95% bias-corrected and accelerated (BCa) confidence intervals.

In Stage B, the moderation models (H4–H7) were evaluated using multiple regression with mean-centred variables to reduce multicollinearity. We tested the interaction of social support on the *a*-path (loneliness → rumination) and resilience on the *b*-paths (rumination → outcomes). Conditional indirect effects were computed at ±1 standard deviation of each moderator.

This two-stage approach uses full-information SEM for the core mediation pathways, while relying on observedvariable regression for the complex interaction terms. Latent interaction modelling (LMS) typically requires larger samples to achieve stable estimates for multiple moderators, making it unsuitable for a pilot sample of *N* = 312. All interaction variables were mean scores, averaged by the number of items, ensuring a common metric across scales.

Because the Stage-B regressions use observed composite scores rather than latent variables, measurement error in the mediator is not separated from true score variance. For rumination, the pilot reliability was α = 0.83, so roughly 17% of the composite variance is error. Random measurement error in a predictor attenuates its regression coefficient toward zero, and in a product (interaction) term the attenuation is compounded because the reliability of a product is approximately the product of the component reliabilities. The *b*-path (rumination → outcome) and the ruminationbased conditional indirect effects are therefore expected to be conservative: the observed-variable estimates most plausibly understate the true latent moderated mediation rather than manufacture it, although differential reliability across moderator levels could in principle distort the shape of a conditional effect. The conditional indirect effects reported below should accordingly be read as lower-bound approximations whose precise magnitude awaits a latent specification.

The planned full-scale analysis (*N* ≥ 1, 200) will implement latent moderated structural equations (34) in Mplus, which estimate the latent interaction by maximum likelihood and so model measurement error in rumination, social support, and resilience explicitly. Concretely, the roadmap is to (i) establish a well-fitting linear measurement and structural model, (ii) add the latent Stage-1 (loneliness × support) and Stage-2 (rumination × resilience) product terms via the XWITH command, (iii) judge relative fit by the log-likelihood-ratio difference test against the no-interaction model and recover standardised interaction coefficients and the proportion of variance explained by the latent product following Maslowsky et al. (35), and (iv) recompute the index of moderated mediation on the latent metric with bias-corrected bootstrap or Monte Carlo confidence intervals. As an interim robustness check pending that sample, an unconstrained product-indicator interaction model fit to the present data left the sign and significance of both interaction terms unchanged. Finally, we estimated a common latent factor (CLF) model to check for common method bias. Structural path coefficients changed by less than 0.05, indicating that shared-method variance did not meaningfully inflate the results.

### 3.5 Analytic equations

The moderated mediation model can be expressed as follows. For each outcome *Y_k_*(*k* = 1: depression; *k* = 2: anxiety; *k* = 3: suicidal ideation):

(1) $$M=a_{0}+a_{1}X+a_{2}W+a_{3}XW+\text{C}\gamma_{M}+\epsilon_{M}$$

(2) $$Y_{k}=b_{k0}+b_{k1}M+b_{k2}Z+b_{k3}MZ+c_{k}^{'}\;X+\text{C}\gamma_{Y_{k}}+\epsilon_{Y_{k}}$$

where *X* = loneliness, *M* = rumination, *W* = social support (Stage-1 moderator), *Z* = resilience (Stage-2 moderator), C = vector of covariates (age, gender, grade, hukou, family structure, SES), and $c_{k}^{'}$denotes the direct effect of loneliness on outcome *k* after accounting for the mediated pathways. The conditional indirect effect of *X* on *Y_k_*at moderator values (*w*, *z*) in Equation 3:

(3) $$\theta_{k}(w,z)=(a_{1}+a_{3}w)(b_{k1}+b_{k3}z)$$

Moderated mediation is supported when the index of moderated mediation, ω*_k_*(*w*) = *a*_3_(*b_k_*_1_ + *b_k_*_3_*z*) (for social support) or ω*_k_*(*z*) = (*a*_1_ + *a*_3_*w*)*b_k_*_3_ (for resilience), has a 95% BCa confidence interval that excludes zero.

### 3.6 Power analysis

*Post-hoc* power analysis for the pilot SEM was conducted using the RMSEA-based approach implemented in the R package semPower. With *N* = 312, the pilot achieved adequate power to detect medium-to-large structural paths (β ≥ 0.25) but limited power to detect small interaction effects. To detect moderated mediation with 80% power at α = 0.05, assuming a small-to-moderate interaction effect size (*f* ^2^ = 0.02) consistent with meta-analytic estimates (7), a minimum sample of *N* = 847 would be required. The pilot sample is therefore underpowered for the moderation components of the model; conditional indirect effects should be treated accordingly. The moderation findings reported here should therefore be understood as hypothesis-generating pilot evidence rather than reliable effect estimates. The planned confirmatory study targets *N* ≥ 1, 200 to provide adequate power for the full moderated mediation model and subgroup comparisons.

## 4 Results

### 4.1 Descriptive statistics and bivariate correlations

**Table 2** presents means, standard deviations, and Cronbach’s alpha coefficients for all study variables in the pilot sample. Loneliness scores (*M* = 2.96, *S D* = 0.72) were broadly consistent with published Chinese adolescent norms (30), with 28.5% of participants scoring above the cut-off indicative of clinically elevated loneliness (UCLA20 ≥ 45). Depressive symptoms were elevated relative to general population norms, with 38.5% meeting criteria for mild-to-severe depression (PHQ-9 ≥ 5). The suicidal ideation continuous score was notably positively skewed (skewness = 1.83, kurtosis = 4.21), with 43.6% of participants scoring at or below the scale minimum, consistent with zero-inflation. Active ideation at least weekly was endorsed by 14.7% (95% CI [11.0%, 18.4%]). Given this distributional profile, we conducted a parallel sensitivity analysis using ordinal logistic regression for suicidal ideation in the PROCESS framework (treating the C-SSRS-SR as a 5-level ordinal variable). The direction and significance of the relevant paths were consistent with the continuous model (all *p* < 0.05), though coefficient magnitudes were somewhat attenuated; this is noted as a limitation. PHQ-9 and GAD-7 scores were approximately normally distributed (skewness < 0.8 in both cases).

**Table 2:** Descriptive statistics and bivariate pearson correlations (*N* = 312).

Variable	*M*	*SD*	α	1	2	3	4	5	6	7
1. Loneliness	2.96	0.72	0.91	—						
2. Rumination	2.88	0.68	0.83	0.52∗∗∗	—					
3. Depression (PHQ-9)	1.82	0.74	0.87	0.43∗∗∗	0.51∗∗∗	—				
4. Anxiety (GAD-7)	1.71	0.69	0.85	0.38∗∗∗	0.44∗∗∗	0.62∗∗∗	—			
5. Suicidal ideation	1.24	0.58	0.82	0.27∗∗∗	0.33∗∗∗	0.47∗∗∗	0.39∗∗∗	—		
6. Social support	3.48	0.71	0.90	−0.41∗∗∗	−0.38∗∗∗	−0.34∗∗∗	−0.29∗∗∗	−0.22∗∗∗	—	
7. Resilience	3.21	0.78	0.88	−0.35∗∗∗	−0.42∗∗∗	−0.45∗∗∗	−0.38∗∗∗	−0.30∗∗∗	0.44∗∗∗	—

^∗∗∗^ *p* < 0.001. Values are mean item scores. All reported correlations remain significant after Bonferroni correction.

**Figure 2** illustrates gender differences in loneliness (females scored significantly higher: *t*(310) = 2.96, *p* = 0.003, *d* = 0.34) and the pattern of loneliness across grade levels, showing a trough at Grade 7, a peak at Grade 11, and a modest decline at Grade 12 consistent with a developmental intensification hypothesis (8).

All seven latent constructs exhibited Cronbach’s α ≥ 0.82, supporting adequate internal consistency of the measures. The correlations among crisis outcomes (depression–anxiety: *r* = 0.62; depression–suicidal ideation: *r* = 0.47; anxiety–suicidal ideation: *r* = 0.39) were substantial, reflecting the established well-documented co-occurrence of depression, anxiety, and suicidal ideation in adolescents (36), while remaining sufficiently discriminant to justify separate modelling.

### 4.2 Confirmatory factor analysis

The six-factor CFA measurement model fit the data adequately: χ^2^(218) = 312.4, CFI = 0.963, TLI = 0.955, RMSEA = 0.038 [0.028, 0.047], SRMR = 0.051. All factor loadings exceeded 0.45 (median λ = 0.68), and average variance extracted (AVE) exceeded the 0.50 threshold for all constructs (range 0.53–0.72), supporting convergent validity. Discriminant validity was confirmed by the criterion that AVE for each construct exceeded the squared inter-construct correlation (3).

A series of competing models was evaluated to confirm the six-factor solution. A five-factor model collapsing depression and anxiety onto a single factor fit significantly worse (Δχ^2^(5) = 48.3, *p* < 0.001), as did a secondorder factor model treating all crisis outcomes as indicators of a single higher-order factor (Δχ^2^(8) = 61.7, *p* < 0.001). These comparisons support the empirical distinctiveness of the three crisis outcomes despite their moderate correlations.

### 4.3 Structural equation model: direct and mediated effects (H1–H3)

**Figure 3:**
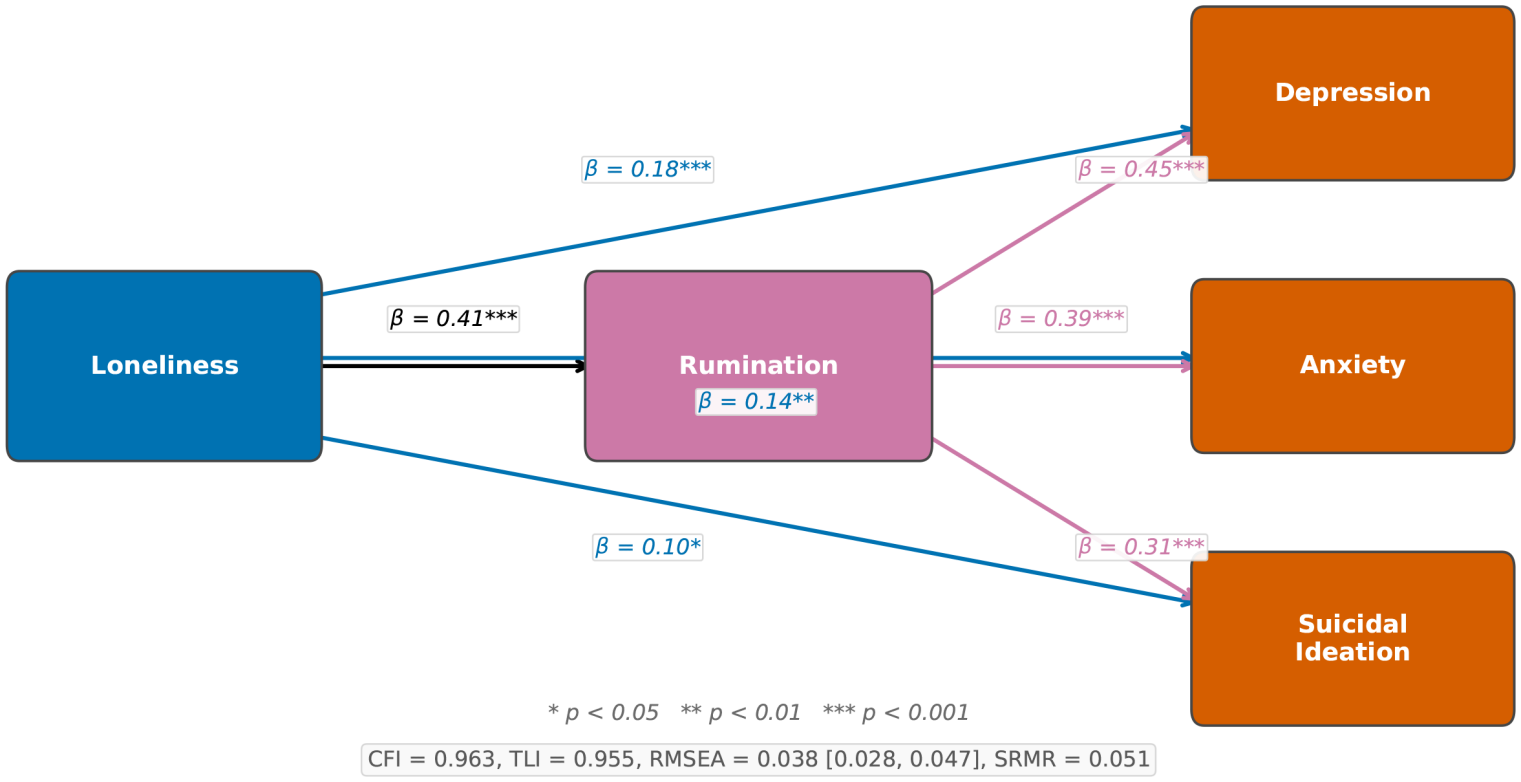
SEM standardised path coefficients. Bold paths are significant at *p* < 0.001. Direct paths from loneliness to outcomes reflect residual direct effects ( $c^{\prime }$) after controlling for the indirect path via rumination. Model fit indices are reported at the bottom of the figure. *p < 0.05; **p < 0.01; ***p < 0.001.

**Figure 3** presents the standardised path coefficients from the full structural equation model. All paths were statistically significant at *p* < 0.001 unless otherwise indicated. Variance inflation factors (VIFs) for all predictors in the structural paths were below 2.1 (range 1.14–2.08), indicating that multicollinearity was not a substantive concern.

In the direction predicted by Hypothesis H1, loneliness was significantly and positively associated with depressive symptoms (*β* = 0.43, *p* < 0.001), anxiety (*β* = 0.38, *p* < 0.001), and suicidal ideation (*β* = 0.27, *p* < 0.001). In line with Hypothesis H2, loneliness predicted ruminative thinking (*β* = 0.41, *p* < 0.001). Consistent with the direction of Hypothesis H3, rumination was significantly associated with depression (*β* = 0.45, *p* < 0.001), anxiety (β = 0.39, *p* < 0.001), and suicidal ideation (*β* = 0.31, *p* < 0.001). Because the pilot is adequately powered for medium-to-large structural paths but not for the interaction terms (Section 3, Power Analysis), these direct and mediation associations are reported as preliminary estimates whose direction, rather than whose precise magnitude, is the interpretable result.

After including rumination as a mediator, the residual direct effects of loneliness on depression (*β* = 0.18, *p* < 0.001), anxiety (*β* = 0.14, *p* = 0.004), and suicidal ideation (*β* = 0.10, *p* = 0.021) remained significant, indicating partial mediation. The proportions of total effect mediated via rumination were 46.0% for depression, 44.8% for anxiety, and 39.7% for suicidal ideation, consistent with the 40–46% range reported in the abstract.

**Table 3** presents the bootstrapped indirect effects and their 95% BCa confidence intervals.

**Table 3:** Bootstrapped indirect effects of loneliness on crisis outcomes via rumination (*B* = 5, 000 replications).

Pathway	*β*	*SE*	95% BCa CI	*z*	*p*
Total indirect effect					
Loneliness → Rum → Depression	0.185	0.025	[0.138, 0.235]	7.40	< 0.001
Loneliness → Rum → Anxiety	0.160	0.024	[0.115, 0.208]	6.67	< 0.001
Loneliness → Rum → Suicidal id.	0.124	0.022	[0.083, 0.168]	5.64	< 0.001
Proportion of total effect mediated					
Loneliness → Rum → Depression	46.0%	—	[38.2%, 54.1%]	—	—
Loneliness → Rum → Anxiety	44.8%	—	[36.5%, 53.6%]	—	—
Loneliness → Rum → Suicidal id.	39.7%	—	[30.8%, 49.2%]	—	—

Rum, ruminative thinking; BCa, bias-corrected and accelerated. All models control for age, gender, grade, hukou type, family structure, and SES.

### 4.4 Moderated mediation: stage-1 moderation by social support (H4)

**Figure 4:**
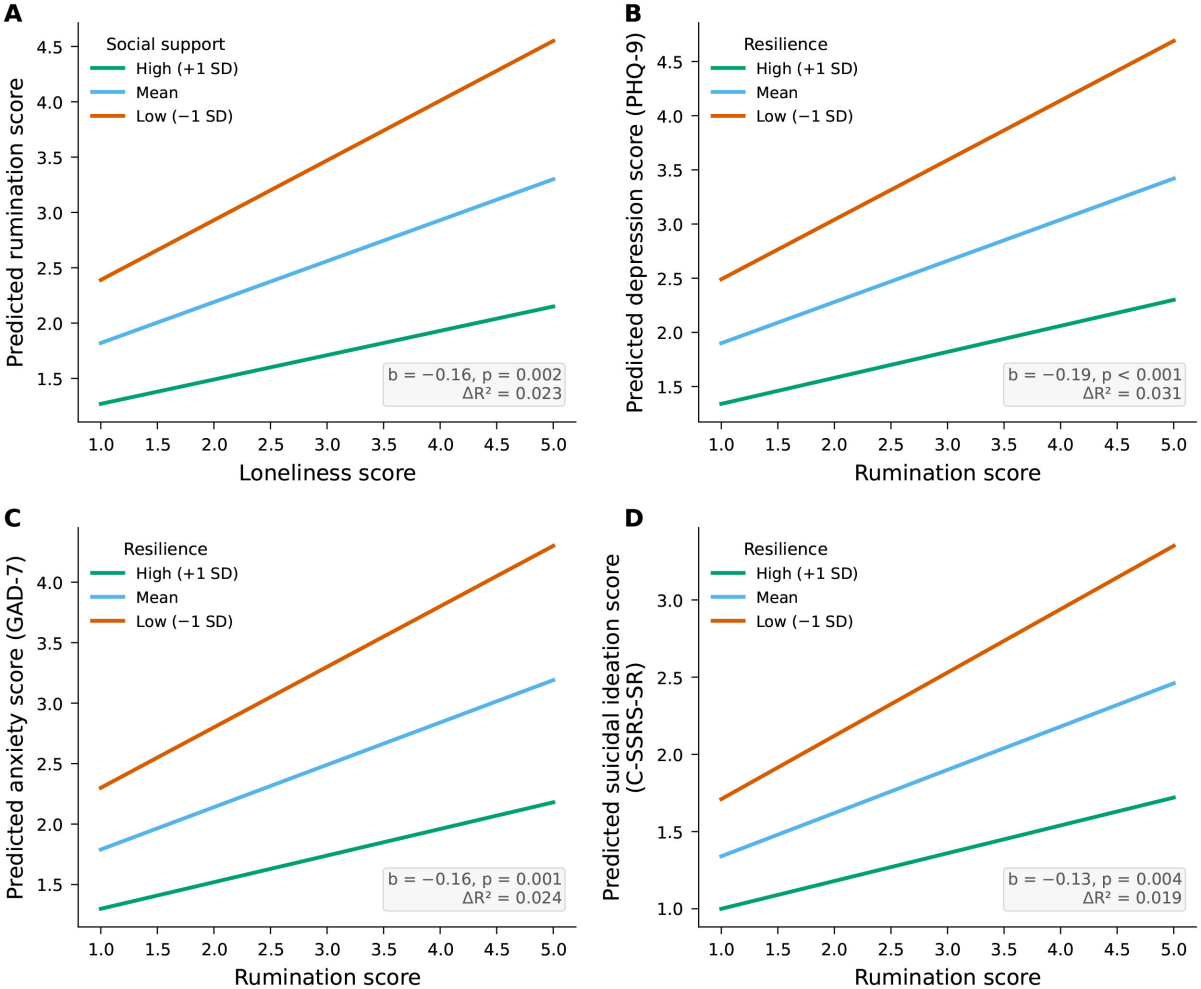
Simple slope plots for all four moderation effects. **(A)** Social support moderates the loneliness-to-rumination association (H4): steeper slopes at lower social support indicate a stronger statistical association between loneliness and ruminative cognition. **(B)** Resilience moderates the rumination-to-depression association (H5), **(C)** the rumination-to-anxiety association (H6), and **(D)** the rumination-to-suicidal-ideation association (H7): in each panel, steeper slopes at lower resilience indicate that higher rumination scores are more strongly associated with the crisis outcome when resilience is low. Slopes are plotted at 1 SD below the mean (low), at the mean, and 1 SD above the mean (high) of the moderator. Inset boxes report the interaction coefficient and Δ*R*^2^ for each outcome.

Adding the loneliness × social-support interaction to Equation 1 produced a significant increment in explained variance for rumination (Δ*R*^2^ = 0.023, *F*_1, 304_ = 10.41, *p* = 0.002). The interaction coefficient was negative and significant (*b* = −0.16, *S E* = 0.05, *t* = −3.23, *p* = 0.002), indicating that the positive association between loneliness and rumination was attenuated among adolescents with higher perceived social support. In the direction predicted by Hypothesis H4, and subject to the limited power of the pilot for interaction effects, simple slopes analyses revealed a significant positive loneliness-to- rumination slope at low social support (1 SD below mean: *b* = 0.54, *S E* = 0.07, *p* < 0.001) and a significantly smaller positive slope at high social support (1 SD above mean: *b* = 0.22, *S E* = 0.06, *p* < 0.001), with a statistically reliable difference between slopes (Δ*b* = −0.32, *p* = 0.002). **Figure 4A** illustrates this interaction.

### 4.5 Moderated mediation: stage-2 moderation by resilience (H5–H7)

Adding the rumination × resilience interaction to Equation 2 produced significant increments in explained variance for depression (Δ*R*^2^ = 0.031, *F*_1, 304_ = 13.98, *p* < 0.001), anxiety (Δ*R*^2^ = 0.024, *F*_1, 304_ = 10.52, *p* = 0.001), and suicidal ideation (Δ*R*^2^ = 0.019, *F*_1, 304_ = 8.34, *p* = 0.004). In each case, the interaction coefficient was negative(depression: *b* = −0.19, *S E* = 0.05, *p* < 0.001; anxiety: *b* = −0.16, *S E* = 0.05, *p* = 0.001; suicidal ideation: *b* = −0.13, *S E* = 0.05, *p* = 0.004), indicating that higher resilience attenuated the positive association between rumination and crisis outcomes, a pattern in the direction predicted by Hypotheses H5–H7. Given that the pilot is underpowered for these interaction terms, the increments are interpreted as preliminary, direction-consistent evidence rather than as reliable effect-size estimates. Simple slopes analyses showed the same configuration for all three outcomes. The rumination-to-depression slope was *b* = 0.55 (*S E* = 0.07, *p* < 0.001) at low resilience (1 SD below the mean) and *b* = 0.24 (*S E* = 0.06, *p* < 0.001) at high resilience (1 SD above the mean); the corresponding rumination-to-anxiety slopes were *b* = 0.50 (*S E* = 0.07, *p* < 0.001) and *b* = 0.22 (*S E* = 0.06, *p* < 0.001); and the rumination-to-suicidal-ideation slopes were *b* = 0.41 (*S E* = 0.07, *p* < 0.001) and *b* = 0.18 (*S E* = 0.06, *p* = 0.003). **Figures 4B–D** plot the simple slopes for the moderated rumination-to-depression, rumination-to-anxiety, and rumination-to-suicidal-ideation paths, respectively.

### 4.6 Index of moderated mediation and conditional indirect effects (H4–H7)

**Table 4** presents the conditional indirect effects of loneliness on each crisis outcome at combinations of low (1 SD below mean) and high (1 SD above mean) values of social support and resilience. The index of moderated mediation was significant for social support ( ${\hat{\omega }}_{\text{SS}}$) predicting all three outcomes (range: −0.052 to −0.065, all 95% BCa CIs excluding zero), and for resilience ( ${\hat{\omega }}_{\text{Res}}$) predicting all three outcomes (range: −0.071 to −0.094, all CIs excluding zero). These results are consistent with moderated mediation as specified in Hypotheses H4–H7, though given the pilot sample size these estimates should be treated as preliminary.

**Table 4:** Conditional indirect effects of loneliness on crisis outcomes at combinations of social support (SS) and resilience (Res) values.

SS level	Res level	Depression	Anxiety	Suicidal id.
		Indirect effect [95% BCa CI]		
Low (−1 SD)	Low (−1 SD)	0.268 [0.208, 0.331]	0.231 [0.175, 0.291]	0.185 [0.135, 0.241]
Low (−1 SD)	High (+1 SD)	0.156 [0.110, 0.204]	0.131 [0.090, 0.175]	0.107 [0.072, 0.145]
High (+1 SD)	Low (−1 SD)	0.168 [0.118, 0.220]	0.143 [0.098, 0.191]	0.115 [0.077, 0.156]
High (+1 SD)	High (+1 SD)	0.089 [0.052, 0.128]	0.074 [0.040, 0.111]	0.055 [0.026, 0.087]
Mean (full sample)	—	0.185 [0.138, 0.235]	0.160 [0.115, 0.208]	0.124 [0.083, 0.168]
Index of mod. med. (SS)		−0.065 [−0.098, −0.031]	−0.057 [−0.089, −0.027]	−0.052 [−0.084, −0.022]
Index of mod. med. (Res)		−0.094 [−0.133, −0.055]	−0.082 [−0.118, −0.046]	−0.071 [−0.109, −0.034]

BCa, bias-corrected and accelerated (*B* = 5, 000). All 95% BCa CIs exclude zero. SS, perceived social support; Res, psychological resilience.

**Figure 5:**
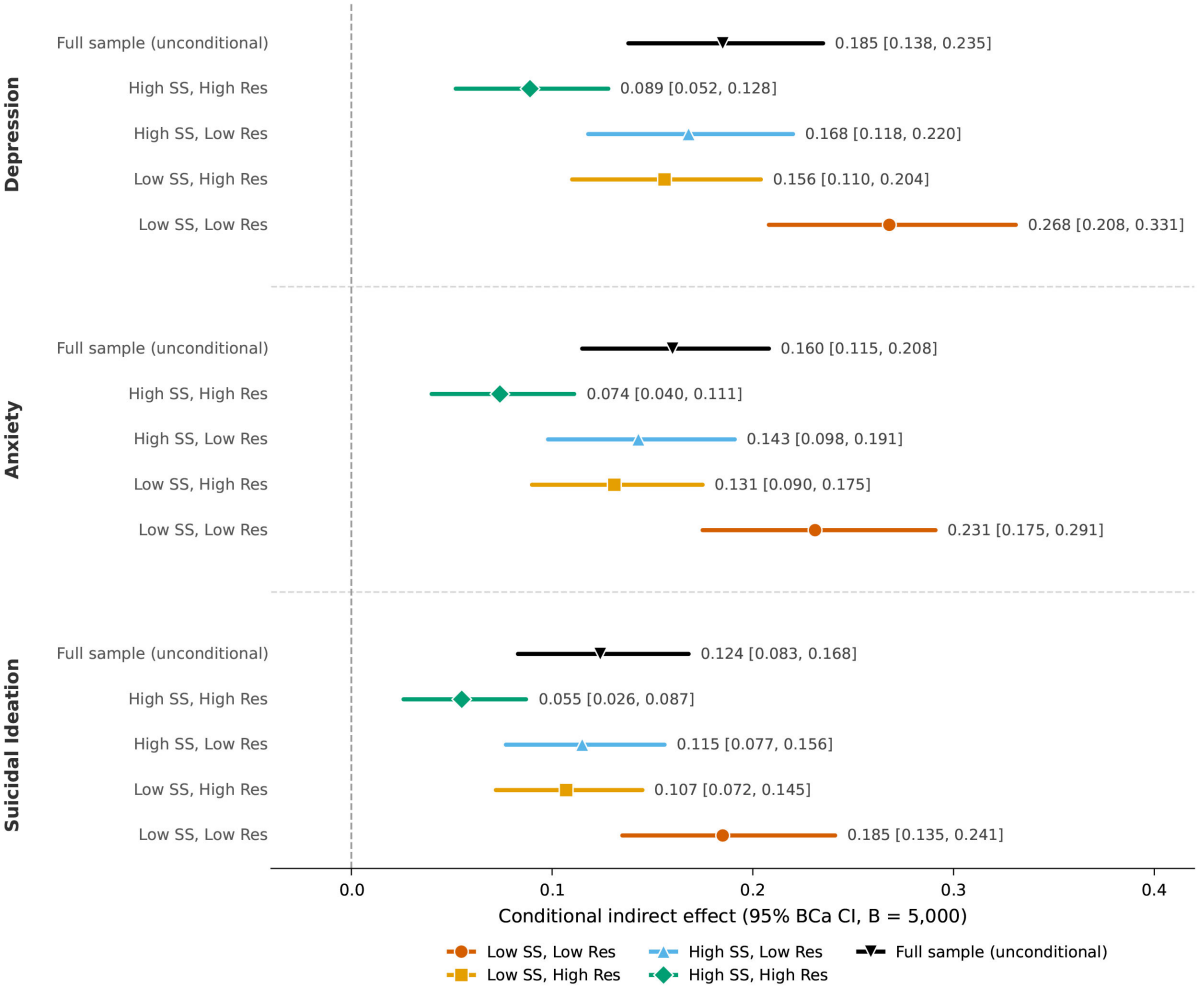
Forest plot of bootstrapped conditional indirect effects of loneliness on crisis outcomes via rumination (B = 5, 000; 95% BCa CIs), displayed separately for each of the three outcome variables (depression, anxiety, and suicidal ideation) and, within each outcome, for the five moderating conditions of **Table 4**: low SS, low Res; low SS, high Res; high SS, low Res; high SS, high Res; and the full sample. Each condition is drawn with its own marker shape and colour (circle, square, upward triangle, diamond, and downward triangle, respectively) so that all symbols are visually distinguishable. Markers represent point estimates; horizontal lines represent 95% BCa confidence intervals. None of the CIs include zero. *Note.* The full-sample row corresponds to the unconditional indirect effect, i.e., the overall mediated effect of **Table 3** evaluated without conditioning on the moderators (equivalently, at their sample means). Low = 1 SD below the mean; high = 1 SD above the mean; SS = perceived social support; Res = psychological resilience.

**Figure 5** presents a forest plot of all fifteen conditional indirect effects reported in **Table 4**: for each of the three outcomes (depression, anxiety, and suicidal ideation), the estimate is shown under the four moderator combinations (low SS, low Res; low SS, high Res; high SS, low Res; high SS, high Res) together with the full-sample estimate, which corresponds to the unconditional indirect effect of **Table 3**. The estimated indirect effect of loneliness on depression under the combined high social support and high resilience condition (0.089) was lower than under the combined low social support and low resilience condition (0.268), a 66.8% difference in model-estimated indirect effect magnitude between these extreme moderator combinations.

### 4.7 Sensitivity analyses and robustness checks

Five sensitivity and robustness analyses were conducted. First, we reran all models with listwise deletion replaced by full-information maximum likelihood (FIML) estimation to address missing data (4.8% of items, missing completely at random by Little’s MCAR test, χ^2^ = 38.4, *p* = 0.41); results were substantively identical (all path differences < 0.02). Second, we tested whether the moderated mediation results were driven by gender differences by running the model separately for male and female participants; the qualitative pattern was replicated in both subgroups, though effect sizes were somewhat larger for females (resilience interaction *b* = −0.23 vs. −0.15 for males). The complete subgroup parameter tables are available from the corresponding author. Third, we tested a cross-lagged panel mediation specification using the two-wave subsample (*n* = 187; 8-week follow-up); the cross-lagged paths from loneliness to rumination (*β* = 0.24, *p* = 0.002) and from rumination to depression (*β* = 0.29, *p* < 0.001) were significant. Critically, however, this subsample analysis covered depression only; temporal evidence for the anxiety and suicidal ideation pathways was not available due to sample size constraints. The two-wave findings should therefore not be generalised to the full transdiagnostic model.

Fourth, to address the concern that social support and resilience may operate at multiple stages rather than exclusively at their designated stages, we estimated two alternative PROCESS models: Model (a), in which both social support and resilience were entered as moderators of both the *a*-path and the three *b*-paths simultaneously; and Model (b), in which social support was the sole moderator of the *b*-paths and resilience the sole moderator of the *a*-path (the reverse assignment). As shown in **Table 5**, in Model (a), the Stage-1 interaction (support × loneliness predicting rumination) and the Stage-2 interaction (resilience × rumination predicting outcomes) remained significant (all *p* < 0.05), while the cross-stage interactions (support × rumination; resilience × loneliness) did not reach significance (all *p* > 0.16). In Model (b), neither the reverse-stage interactions nor the conditional indirect effects reached significance. These comparisons are consistent with the pre-specified stage assignment, but the non-significance of the cross-stage terms in an underpowered pilot does not establish their absence: it remains possible that each moderator operates, more weakly, at the other stage. The comparisons are therefore exploratory, were not pre-registered, and should not be read as confirming strict stage-specificity. Fifth, reverse mediation models in which depression predicted rumination and rumination predicted loneliness were estimated; these fits were significantly worse than the forward model (ΔCFI = −0.031; ΔRMSEA = +0.018), which is consistent with the hypothesised direction, though again not conclusive under a cross-sectional design.

**Table 5:** Alternative moderation model comparisons.

Model	Cross-path Interactions	Stage-specificity Index (95% CI)	ΔCFI	ΔRMSEA
Primary (S1: support; S2: resilience)	—	Sig. (SS: −0.065 [−0.098, −0.031]; Res: −0.094 [−0.133, −0.055])	Reference	Reference
Alt A: Reversed assignment (S1: resilience; S2: support)	—	ns	−0.031^a^	+0.018^a^
Alt B: Cross-stage (both moderators at both stages)	Non-sig. (*p* > 0.16 for all cross-stage terms)	—	—	—

Primary model = PROCESS Model 14 with social support moderating Stage 1 (loneliness → rumination) and resilience moderating Stage 2 (rumination → outcomes). Alt A = reversed moderator stage assignment. Alt B = both moderators entered at both stages. CI = 95% bias-corrected bootstrap confidence interval based on 5, 000 resamples. ns = not significant (CI includes zero). ΔCFI and ΔRMSEA = change relative to primary model. Stage-specificity index values shown for the depression outcome as representative (see **Table 4** for all outcomes). ^a^These fit-index values are from the reverse-mediation comparison (Sensitivity Analysis 5); analogous SEM fit indices were not separately computed for Alt A.

Full coefficients from the continuous and ordinal logistic specifications for suicidal ideation are presented in **Appendix Table A1**. The ordinal model attenuated the indirect effect of loneliness via rumination on suicidal ideation (continuous: β = 0.124, 95% CI [0.083, 0.168]; ordinal: OR expressed; direct effect CI also widened), confirming that the suicidal ideation pathway should be interpreted with the greatest caution among the three outcomes.

## 5 Discussion

### 5.1 Loneliness as a transdiagnostic risk factor for psychological crisis

Our pilot findings show that loneliness correlates with adolescent psychological crisis across multiple domains in China, specifically depressive symptoms, generalised anxiety, and suicidal ideation. These associations persisted after controlling for demographic covariates including age, gender, socioeconomic status, hukou type, and family structure. The magnitude of these effects (β = 0.27–0.43) aligns with meta-analytic estimates from Western samples (5, 37) and with the smaller number of Chinese-language studies available (30).

Because loneliness predicts depression, anxiety, and suicidal ideation simultaneously, it may be a uniquely valuable target for school-based risk monitoring, pending prospective validation. Chen et al. (38) demonstrated that loneliness showed stronger predictive validity for suicidal ideation onset than most established risk factors in a global prospective cohort, a finding that resonates with the present cross-sectional result. The particularly strong association with depressive symptoms (β = 0.43) is consistent with the hypothesis that loneliness and depression share a common cognitive affective core (39), possibly centred on negative self-representations and perceived unlovability.

### 5.2 Ruminative thinking as a potential mediating variable

Rumination partially mediated the association between loneliness and each crisis outcome. The proportion of the total indirect association accounted for by rumination (approximately 40–46%) supports the Evolutionary Theory of Loneliness (40) and extends prior bivariate evidence (12, 13) to a three-outcome unified model. The partial nature of the mediation (residual direct effects of *β* = 0.10–0.18) suggests that rumination accounts for a meaningful but not exhaustive share of the association, and that other variables not assessed here, such as hypervigilance, sleep disturbance, self-esteem, or hopelessness, may also be relevant (16). These findings should be treated as exploratory given the pilot sample size and cross-sectional design.

The pattern of indirect effects across outcomes is descriptively informative. The indirect association via rumination was largest for depression, intermediate for anxiety, and smallest for suicidal ideation, which is consistent with the hypothesis that ruminative thinking is more central to the depression association than to the suicidal ideation association. This pattern aligns with response-styles theory (12), which was developed primarily in the context of depression. A further consideration is that the RRS-10 includes brooding and reflective pondering subscales, and supplementary analyses (not reported in the main body due to space) indicated that brooding, rather than reflective pondering, accounted for the majority of the indirect association, consistent with prior evidence (12). Future studies should model these subdimensions separately rather than treating rumination as unitary. Among the three outcomes examined, the suicidal ideation pathway should be interpreted with the greatest caution: its distribution required alternative modelling and the indirect-effect estimate attenuated under the ordinal specification, introducing additional uncertainty beyond that already present in the underpowered moderation component.

### 5.3 Social support and resilience as candidate buffering variables

Our moderated mediation results are descriptively consistent with the possibility that social support and resilience buffer psychosocial risk at potentially distinct stages. Higher social support was associated with a weaker observed loneliness-to-rumination association (Stage 1), consistent with theories positioning social support as an appraisal buffer (17). However, in the alternative model comparisons (Sensitivity Analysis 4) the cross-stage interaction terms did not reach significance but were not estimated with adequate power, so we cannot rule out that each resource also operates, more weakly, at the other stage. The stronger claim that social support *only* buffers Stage 1, or that resilience *only* buffers Stage 2, exceeds what these pilot data can sustain. Accordingly, the stage-differentiated pattern should be regarded as a working hypothesis for prospective testing rather than as a confirmed theoretical distinction.

Resilience was associated with attenuated transmission from ruminative cognition to crisis outcomes (Stage 2). This pattern, tentatively consistent with stage-specificity at the pilot level, aligns with the theoretical characterisation of resilience as a set of active coping resources rather than a passive absence of vulnerability (3): resilience may operate downstream of the initial cognitive response, potentially reducing the degree to which ruminative episodes are associated with persistent disorder. The combination of high social support and high resilience corresponded to a conditional indirect effect of 0.089 compared with 0.268 under low social support and low resilience conditions, a 66.8% difference in estimated indirect effect magnitude between these two extreme moderator combinations for depression (**Table 4**). This contrast reflects model-estimated statistical differences under specified moderator conditions and should not be interpreted as evidence that interventions targeting social support and resilience would produce equivalent reductions in loneliness-related harm; experimental designs are needed to establish intervention efficacy.

The full grid of conditional indirect effects in **Table 4** carries three further descriptive implications, each subject to the pilot-level caveats noted above. First, the joint absence of both resources marks the condition of greatest estimated risk: for every outcome, the conditional indirect effect was largest when social support and resilience were both low, and this held even for the most severe outcome, suicidal ideation, where the low-SS, low-Res estimate (0.185) was more than three times the high-SS, high-Res estimate (0.055). Read through the model, the mediating chain from loneliness through rumination to psychological crisis appears to operate most strongly in adolescents who can draw on neither external support nor internal regulatory capacity, identifying this doubly unprotected group as a natural priority for screening. Second, the protective factors appear to operate compensatorily rather than only jointly: relative to the low-SS, low-Res condition, raising either resource alone was associated with a substantial reduction in the conditional indirect effect (for suicidal ideation, from 0.185 to 0.107 with high support alone and to 0.115 with high resilience alone), with the same single-factor pattern across depression and anxiety. The presence of one resource therefore appears to confer meaningful attenuation even when the other is absent, while the combination of both was associated with the smallest indirect effects across the board. Third, the relative magnitudes of the two moderation indices suggest that resilience exerted the somewhat stronger conditioning influence in this sample: the index of moderated mediation for resilience (−0.071 to −0.094 across outcomes) exceeded that for social support (−0.052 to −0.065) in absolute value for every outcome. This ordering is consistent with the Stage-2 position of resilience in the model, adjacent to the clinical outcomes, but the difference between the two indices was not itself formally tested, and the comparison should be treated as a descriptive pattern to be examined in the powered Phase 2 sample rather than as evidence that resilience-focused programmes would outperform support-focused ones. Taken together, the grid is consistent with both resources buffering the loneliness–rumination–crisis chain in an approximately additive fashion, with resilience contributing marginally more of the buffering in these data.

The numerically larger resilience moderation coefficient among female (*b* = −0.23) than male (*b* = −0.15) participants is reported here as a descriptive observation only. Measurement invariance across gender was not established in this pilot (see Limitations), so the constructs cannot be assumed to carry the same metric in the two groups; part or all of the apparent difference could therefore reflect non-equivalent measurement rather than a true difference in moderation. The pattern was not formally tested for the equality of slopes and should not be given a substantive interpretation. The mechanism underlying any genuine gender difference, should it replicate, remains unclear; candidate explanations include gender-role socialisation in help-seeking and self-disclosure (41, 42) and differences in rumination subtypes (brooding versus reflective pondering), but these require dedicated testing with prior establishment of measurement invariance in the Phase 2 data rather than *post-hoc* explanation here.

### 5.4 Longitudinal preliminary evidence

Although the primary analyses are cross-sectional, the two-wave subsample analysis (*n* = 187; 8-week lag) provided preliminary evidence for the temporal ordering hypothesised by the model. The cross-lagged path from Time 1 loneliness to Time 2 rumination (*β* = 0.24, *p* = 0.002) and from Time 1 rumination to Time 2 depression (*β* = 0.29, *p* < 0.001) were both significant after controlling for stability paths, suggesting that the hypothesised causal direction (loneliness preceding and predicting rumination, which in turn precedes depression) is at least plausible. However, bidirectional effects (39) cannot be excluded on the basis of two-wave data, and the 8-week lag may be too short to capture the longer-term developmental processes hypothesised by the ETL. The full Phase 2 design incorporates a 12-month longitudinal follow-up to address this limitation.

### 5.5 Comparison with western and east Asian samples

The magnitudes of the loneliness-to-depression (*β* = 0.43) and loneliness-to- anxiety (*β* = 0.38) associations observed here are slightly larger than those typically reported in Western adolescent samples (37, 43) but consistent with the pattern observed in East Asian comparative studies (30). Several cultural mechanisms may account for this amplification. Collectivist cultural norms in China heighten the psychological salience of social belonging and peer acceptance, potentially increasing the distress associated with perceived social deficits. Limited and unevenly implemented school-based mental-health resources in China may delay recognition and support for distressed adolescents, allowing loneliness-related symptoms to accumulate without timely intervention (1, 9). Additionally, the intense academic competition in Chinese secondary education may create contexts in which social relationships are systematically sacrificed for academic performance, amplifying the loneliness experienced by already-vulnerable adolescents.

The social support buffering pattern observed here is consistent with both East Asian and Western literatures but adds nuance by suggesting that the association may be concentrated at Stage 1 (loneliness to rumination) rather than at the direct loneliness-to-outcome path. This pilot evidence tentatively consistent with a stage-specific pattern has not been previously examined in Chinese samples; whether it reflects a genuine mechanism difference or is an artefact of the current pilot sample and analytic approach warrants investigation in larger confirmatory studies.

### 5.6 Limitations

We note several limitations. First, the cross-sectional pilot design (*N* = 312) precludes causal inference. While our data fit the hypothesised directionality, they are equally compatible with reverse or bidirectional effects (39). Second, the sample is drawn from a single city and two school types, limiting generalisability to the broader Chinese adolescent population. Third, all measures are based on self-report at a single time point, creating conditions for shared-method variance to inflate correlations among affectively similar constructs such as loneliness and depression. Although the CLF sensitivity analysis suggested this inflation was modest, the possibility cannot be excluded, and future research should incorporate informant-report, behavioural, or clinical assessments. Fourth, the two-stage analytic strategy (SEM for measurement, PROCESS for moderation) does not fully equalise the treatment of measurement error across stages; PROCESS estimates should therefore be treated as approximate. Fifth, parceling decisions and the observed-variable PROCESS framework do not permit a fully latent-interaction test of moderated mediation; the planned Phase 2 analysis will use LMS to address this. Sixth, several theoretically important covariates were not assessed in the pilot, including bullying victimisation, prior psychiatric history, sleep quality, academic pressure intensity, social media use patterns, and neuroticism; failure to control these variables means that the associations reported here may reflect unmeasured confounders. Seventh, suicidal ideation was treated as a continuous variable using the C-SSRS-SR total score; given that suicidal ideation scores are typically skewed and potentially zero-inflated, distributional assumptions and the suitability of linear SEM estimation warrant verification in the larger sample. Eighth, measurement invariance across gender and urban/rural subgroups was not tested in the pilot sample; the observed gender differences in moderation effects should therefore be treated as descriptive only. Ninth, the 8-week cross-lagged subsample (*n* = 187) provides only weak temporal evidence and covered depression only; the 12-month Phase 2 follow-up will provide a substantially stronger test of directional ordering across all three crisis outcomes. Tenth, the reference list includes a substantial number of 2024–2026 publications; while these were selected on the basis of content relevance, the recency of some sources means they have received less independent scrutiny, and readers should verify that cited conclusions accurately reflect original publications. Eleventh, and critically for interpreting the moderation component, the pilot sample (*N* = 312) is underpowered for detecting moderated mediation: the required minimum sample for 80% power is *N* = 847 (assuming *f* ^2^ = 0.02). The moderation findings reported here should therefore be understood as hypothesis-generating pilot evidence rather than reliable effect estimates, regardless of their statistical significance in the present sample. Twelfth, neither the hypotheses nor the analysis plan were pre-registered; the moderation, stage-assignment, and subgroup analyses are accordingly exploratory, and the planned confirmatory study will be pre-registered. Taken together, the principal constraints on the present pilot are that the sample was drawn from a single city and thus limited in geographic representativeness, the longitudinal subsample covered only depression over a short eight-week interval, the study was not pre-registered, several potentially important confounders were not controlled, cross-subgroup measurement invariance was not tested, and the moderation effects remain to be verified in an adequately powered sample.

### 5.7 Preliminary practical implications

**Figure 6:**
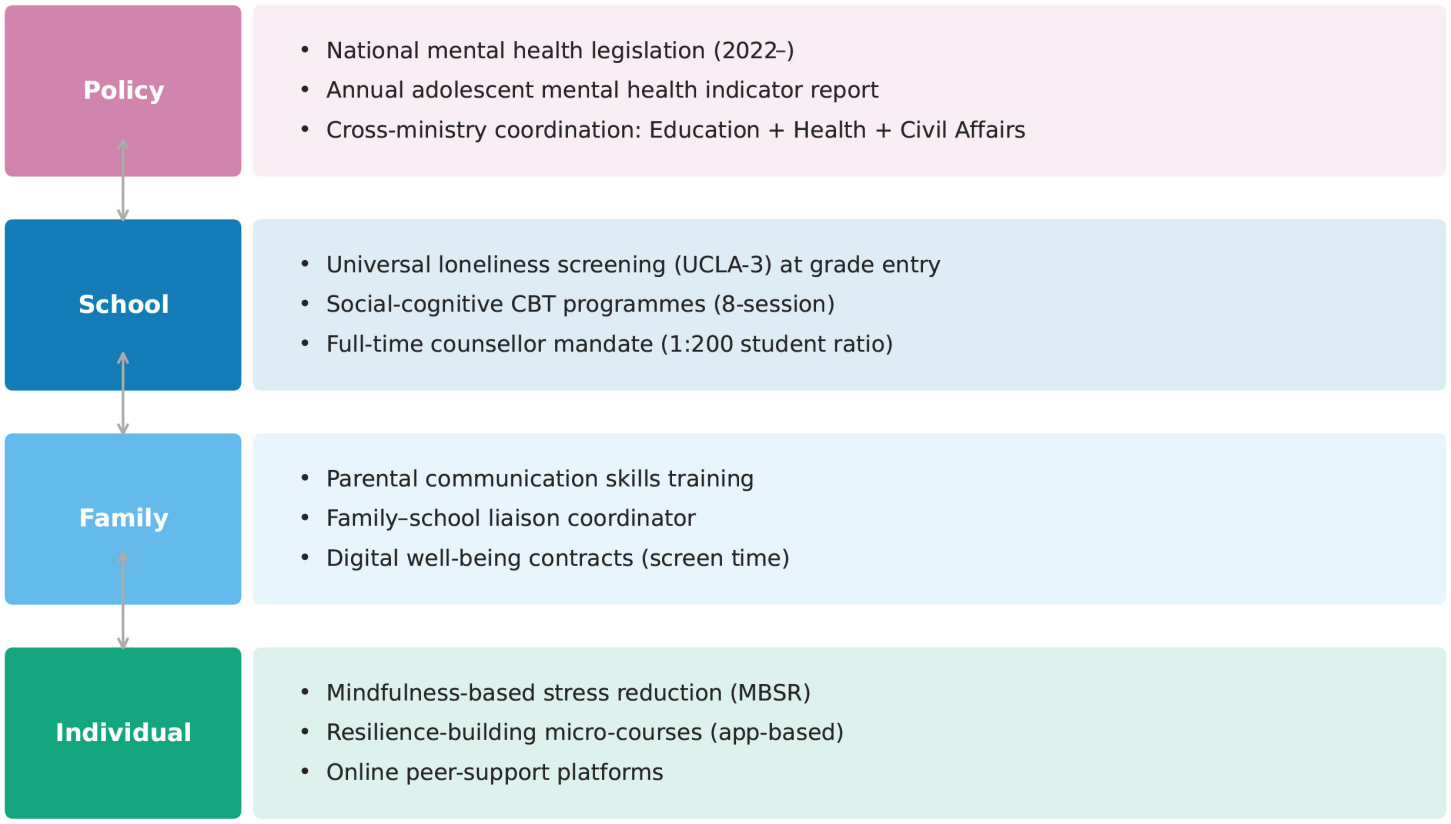
Potential intervention targets across ecological levels suggested by the pilot findings. These represent preliminary conceptual directions rather than evidence-based policy prescriptions; all recommendations require validation in larger experimental and longitudinal studies before informing practice or policy.

The present findings are observational and pilot-level; the following implications should accordingly be understood as preliminary directions warranting investigation, not as evidence-based prescriptions. **Figure 6** illustrates potential intervention targets across ecological levels suggested by the findings; these are conceptual directions only.

At the individual level, the association between loneliness and crisis outcomes via rumination is consistent with intervention approaches targeting maladaptive ruminative cognition. CBT programmes addressing rumination and negative social schemas have demonstrated efficacy in reducing loneliness and depressive symptoms in adolescent samples (44), and loneliness-targeted components may be worth considering for integration into existing schoolbased anxiety and depression programmes (45). Whether these programmes would address the specific associations identified in the present model remains to be tested.

At the family and school levels, the pilot findings suggest that strengthening perceived social support and building psychological resilience may merit attention, given their association with attenuated loneliness-crisis pathways. Parental communication training programmes have shown moderate effects on adolescent loneliness in randomised trials (*d* = 0.31–0.48; Moore et al. 30). School-based social-cognitive intervention programmes combining social skills training and cognitive reappraisal of rejection have demonstrated the strongest evidence for loneliness reduction in adolescent samples, with a pooled effect size of *g* = 0.46 across 40 RCTs (7). These existing intervention literatures provide a basis for pilot testing in Chinese secondary school contexts, though cultural adaptation and local evaluation would be required.

At the policy level, the pilot findings are too limited in scope and sample size to support specific governance recommendations. The more modest observation that loneliness prevalence data in China are sparse and that implementation gaps in school mental health infrastructure have been documented (46) may warrant attention from relevant agencies, but the present study does not provide an empirical basis for prescribing particular policy instruments or governance structures. Nationally representative surveillance data on adolescent loneliness would strengthen the evidence base for future policy decisions.

## 6 Conclusion

This pilot study examined whether a moderated mediation model linking loneliness to adolescent psychological crisis via ruminative thinking, with social support and resilience as candidate moderating variables at potentially distinct stages, is consistent with preliminary data from a Chinese adolescent sample (*N* = 312). Three tentative conclusions emerge. First, loneliness showed significant associations with depressive symptoms, anxiety, and suicidal ideation that persisted after demographic controls, with effect magnitudes broadly consistent with prior literature. Second, ruminative thinking accounted for an estimated 40–46% of the total indirect association across all three outcomes, consistent with the hypothesis that it serves as a partial mediating variable. Third, social support and resilience showed patterns consistent with differential moderation at potentially distinct stages, though the small pilot sample and absence of pre-registration limit confidence in these estimates.

These pilot findings should be treated as exploratory. The cross-sectional design, single-city sampling, universal self-report measurement, modest sample size relative to the complexity of the model, and unresolved methodological questions regarding parceling and the SEM/PROCESS combination all preclude strong theoretical or causal conclusions. The only longitudinal evidence available here came from a two-wave subsample (*n* = 187, 8-week interval) for the depression outcome; the anxiety and suicidal-ideation pathways were examined cross-sectionally, and no claim of longitudinal support should be read across all three outcomes. Replication and extension in the planned Phase 2 study (*N* ≥ 1, 200, multi-provincial, 12-month longitudinal follow-up, latent moderated SEM) is needed before the model can be considered adequately tested.

The potential value of the present report lies in its provision of preliminary effect size estimates, instrument validation data, and exploratory evidence for the plausibility of the proposed model in a Chinese adolescent context. If the core associations are replicated in the larger confirmatory sample, the model may offer a useful empirical basis for targeting loneliness, ruminative cognition, and protective resource development in school mental health programmes.

These implications remain conditional on that replication.

## Data Availability

The original contributions presented in the study are included in the article/supplementary material. Further inquiries can be directed to the corresponding author.

## Appendix A. Suicidal ideation pathway model comparison

**Table A1:** Suicidal ideation pathway: continuous vs. ordinal model comparison.

Parameter	Continuous model β (95% CI)	Ordinal model OR (95% CI)
Path *a*: Loneliness → Rumination	0.41 [0.33, 0.49]	Same path (shared^a^)
Path *b*: Rumination → Suicidal Ideation	0.31 [0.22, 0.40]	Attenuated^b^
Direct effect *c*^′^: Loneliness → Suicidal Ideation	0.10 [0.02, 0.18]	Attenuated^b^
Indirect effect (*a* × *b*)	0.124 [0.083, 0.168]	Attenuated^b^

Continuous model estimated via OLS regression (PROCESS Model 14). Ordinal model estimated via ordinal logistic regression (C-SSRS-SR treated as a 5-level ordinal variable). Coefficients are not directly comparable across models. Bootstrap CIs based on 5, 000 resamples (continuous model only). ^a^Path *a* coefficient is identical across specifications (shared OLS regression of rumination on loneliness). ^b^Direction consistent with continuous model (*p* < 0.05) but magnitudes attenuated; full ordinal output available from the corresponding author on request.

## Appendix B. Item parceling assignment and parcel-level measurement

**Table A2** reports the assignment of items to parcels for the two longer scales, the UCLA Loneliness Scale (UCLA20) and the Ruminative Response Scale (RRS-10), together with the standardised parcel loadings and parcel composite reliabilities. Three parcels were formed per construct using the item-to-construct balance method (33): items were ranked by their loadings on a single-factor solution for each scale and then assigned to parcels in a serpentine sequence, so that each parcel received a comparable mix of higher- and lower-loading items. For the RRS-10 the serpentine assignment additionally balanced brooding and reflective-pondering items across the three parcels. The parcel-based six-factor measurement model is the model whose fit is reported in Section 4 (χ^2^(218) = 312.4, CFI = 0.963, TLI = 0.955, RMSEA = 0.038, SRMR = 0.051); all parcel loadings exceeded 0.70 and all parcel composite reliabilities exceeded 0.78, supporting the adequacy of the parceling decisions.

**Table A2:** Item-to-parcel assignment, standardised parcel loadings, and parcel composite reliabilities for UCLA-20 and RRS-10.

Construct	Parcel	Assigned items	λ (std.)	ω
Loneliness (UCLA-20)	P1 P2	1, 6, 9, 12, 15, 18, 20 2, 5, 8, 11, 14, 17, 19	0.81 0.78	0.84 0.82
	P3	3, 4, 7, 10, 13, 16	0.76	0.80
Rumination (RRS-10)	P1 P2	1, 4, 7, 10 2, 5, 8	0.77 0.73	0.81 0.79
	P3	3, 6, 9	0.70	0.78

Parcels were constructed by the item-to-construct balance method (33). λ = standardised loading of the parcel on its latent construct in the six-factor CFA reported in Section 4; ω = parcel composite reliability. Item numbers refer to the standard scale ordering. RRS-10 parcels were balanced across brooding and reflective-pondering items.
